# IL-11 produced by breast cancer cells augments osteoclastogenesis by sustaining the pool of osteoclast progenitor cells

**DOI:** 10.1186/1471-2407-13-16

**Published:** 2013-01-11

**Authors:** Erin M McCoy, Huixian Hong, Hawley C Pruitt, Xu Feng

**Affiliations:** 1 Department of Pathology, University of Alabama at Birmingham, Birmingham, AL, 35294, USA; 2 Department of Hematology, First Municipal People’s Hospital, Guangzhou Medical College, Guangdong, 510000, China

**Keywords:** Interleukin-11, Breast cancer, Bone metastasis, Osteoclast, Osteolysis, Bone resorption, RANKL

## Abstract

**Background:**

Interleukin (IL)-11, a cytokine produced by breast cancer, has been implicated in breast cancer-induced osteolysis (bone destruction) but the mechanism(s) of action remain controversial. Some studies show that IL-11 is able to promote osteoclast formation independent of the receptor activator of NF-κB ligand (RANKL), while others demonstrate IL-11 can induce osteoclast formation by inducing osteoblasts to secrete RANKL. This work aims to further investigate the role of IL-11 in metastasis-induced osteolysis by addressing a new hypothesis that IL-11 exerts effects on osteoclast progenitor cells.

**Methods:**

To address the precise role of breast cancer-derived IL-11 in osteoclastogenesis, we determined the effect of breast cancer conditioned media on osteoclast progenitor cells with or without an IL-11 neutralizing antibody. We next investigated whether recombinant IL-11 exerts effects on osteoclast progenitor cells and survival of mature osteoclasts. Finally, we examined the ability of IL-11 to mediate osteoclast formation in tissue culture dishes and on bone slices in the absence of RANKL, with suboptimal levels of RANKL, or from RANKL-pretreated murine bone marrow macrophages (BMMs).

**Results:**

We found that freshly isolated murine bone marrow cells cultured in the presence of breast cancer conditioned media for 6 days gave rise to a population of cells which were able to form osteoclasts upon treatment with RANKL and M-CSF. Moreover, a neutralizing anti-IL-11 antibody significantly inhibited the ability of breast cancer conditioned media to promote the development and/or survival of osteoclast progenitor cells. Similarly, recombinant IL-11 was able to sustain a population of osteoclast progenitor cells. However, IL-11 was unable to exert any effect on osteoclast survival, induce osteoclastogenesis independent of RANKL, or promote osteoclastogenesis in suboptimal RANKL conditions.

**Conclusions:**

Our data indicate that a) IL-11 plays an important role in osteoclastogenesis by stimulating the development and/or survival of osteoclast progenitor cells and b) breast cancer may promote osteolysis in part by increasing the pool of osteoclast progenitor cells via tumor cell-derived IL-11. However, given the heterogeneous nature of the bone marrow cells, the precise mechanism by which IL-11 treatment gives rise to a population of osteoclast progenitor cells warrants further investigation.

## Background

Breast cancer is the second leading cause of cancer deaths in women in the United States and this tumor frequently metastasizes to bone. Upon arriving in bone, breast cancer cells disrupt normal bone remodeling by increasing bone resorption, leading to several serious clinical complications including life-threatening hypercalcemia, spinal cord compression, fractures, and extreme bone pain, which result in a significantly decreased quality of life [1,2]. Bone metastases have also been hypothesized to serve as reservoirs for breast cancer to metastasize to other tissues, such as the lung, liver, lymph node, or brain [3]. Thus, breast cancer patients with bone metastases often have a poor prognosis [4].

Breast cancer cells have been shown to promote bone resorption by enhancing osteoclast formation and function via a number of factors derived from the tumor including M-CSF, transforming growth factor (TGF)-β, tumor necrosis factor α, insulin-like growth factor II, parathyroid hormone related peptide, IL-1, IL-6 and IL-11 [2,5-7]. IL-11 is a member of the IL-6 family that recruits a homodimer of gp130, a promiscuous 130 kDa β subunit, after binding to their own non-signaling ligand-specific receptor, IL11R [8,9]. IL-11 is produced by a variety of stromal cells, including fibroblasts, epithelial cells, and osteoblasts and has a variety of functions, including being involved in multiple aspects of hematopoiesis, inhibition of adipocytogenesis, altering neural phenotype, stimulating tissue fibrosis, minimizing tissue injury, and regulating function of chondrocytes, synoviocytes and B cells [10]. Apart from contributing to inflammation, gp130 signaling cytokines also function in the maintenance of bone homeostasis.

Cancer cells have been shown to directly produce IL-11 and to stimulate osteoblasts to secrete IL-11 [11], which in turn is known to suppress the activity of osteoblasts [12]. It has been shown that breast cancer cell lines produce IL-11 [13] and that forced over-expression in cell lines increases tumor burden and osteolytic lesions in an *in vivo* bone metastasis model [5]. Moreover, human breast cancer tumors expressing IL-11 have higher rates of bone metastasis occurrences [3]. Taken together, these observations support the notion that IL-11 plays an important role in breast cancer-induced osteolysis.

Using a knockout mouse model for IL-11, the cytokine was determined to be required for normal bone turnover, with the knockout mice exhibiting increased bone mass as a result of a reduction in osteoclast differentiation [14]. IL-11 has been proposed to stimulate osteoclastogenesis independent of RANKL in one study [15], whereas another study showed that IL-11 did not induce osteoclastogenesis unless marrow cells were co-cultured with calvaria cells [16]. Similarly, other groups argue that IL-11 stimulates osteoblasts to secrete RANKL and/or proteinases [17,18]. Thus, while a functional role of IL-11 in the osteoclastogenic process has been well established, the molecular and cellular mechanisms by which IL-11 promotes osteoclast differentiation and function warrant further investigation. Given the known role of IL-11 in hematopoiesis [10], we hypothesize that IL-11 may exert effects on osteoclast progenitor cells.

In the current study, we further characterize the role of IL-11 in supporting osteoclast formation, function and survival. Our data indicate that IL-11 promotes osteoclastogenesis primarily by increasing the pool of osteoclast progenitor cells. Consistently, we have also found that MDA-MB-231 conditioned media were able to support a population of bone marrow cells that are capable of differentiating into osteoclasts. These findings provide a better understanding of the mechanism by which IL-11 exerts its impact on osteoclast biology, and also suggest a new concept that breast cancer may also promote osteoclast formation by targeting osteoclast progenitor cells.

## Methods

### Chemicals and reagents

Chemicals were purchased from Sigma (St. Louis, MO) unless indicated otherwise. Recombinant GST-RANKL was purified as described previously [19]. Recombinant mouse M-CSF (rM-CSF) (416-ML-010) and IL-11 (418-ML-005) were obtained from R&D Systems (Minneapolis, MN). Neutralizing anti-human IL-11 antibody (AB-218-NA) and normal goat IgG control antibody (AB-108-C) were also obtained from R&D Systems.

### Animals

C57BL/6 mice were purchased from Harlan Industries (Indianapolis, IN). Mice were maintained, and the experiments performed in accordance with the regulations of the University of Alabama at Birmingham (UAB) institutional animal care and use committee (IACUC).

### *In vitro* osteoclastogenesis assays

Breast cancer conditioned α-MEM was prepared by growing the human breast cancer line MDA-MB-231 to confluence, changing media to α-MEM plus 10% inactivated fetal bovine serum (iFBS), and collecting conditioned media after 24 hours. To generate osteoclasts from breast cancer conditioned media- dependent precursors, cells from the bone marrow cavities of the femur and tibia from C56BL/6 mice less than eight weeks of age were used. The bone marrow flushes were maintained in α-MEM for 24 hours at 37°C, in 7% CO_2_, and then cultured in breast cancer conditioned α-MEM or regular α-MEM supplemented with 10% iFBS. Media were changed every 3 days, and after 6 days, cells from the breast cancer conditioned α-MEM pretreated bone marrow flushes were plated in tissue-culture treated dishes at varying densities as indicated specifically in each experiment and treated with rM-CSF (10 ng/ml) and RANKL (100 ng/ml) for 4–6 days to form osteoclasts. Separately, IL-11 neutralizing antibody (5 ug/ml) was added to bone marrow flushes in 20% MDA-MB-231 breast cancer conditioned media, and surviving cells counted at days 3, 4, 5, and 6.

To generate osteoclasts from IL-11-dependant precursors, bone marrow flushes were maintained in α-MEM for 24 hours at 37°C, in 7% CO_2_, and then cultured in α-MEM with the presence of IL-11 (10 ng/ml) or equal volumes of PBS containing 0.01% bovine serum albumin. After 6 days, cells from the IL-11 pretreated bone marrow flushes were plated in tissue-culture treated dishes at varying densities as indicated specifically in each experiment and treated with rM-CSF (10 ng/ml) and RANKL (100 ng/ml) for 4–6 days to form osteoclasts. Separately, IL-11 neutralizing antibody (2 ug/ml) was added to bone marrow flushes in α-MEM with the presence of IL-11 (10 ng/ml), and surviving cells counted at days 3, 4, 5, and 6.

For IL-11 mechanistic studies of osteoclastogenesis, BMMs were isolated from marrow flushes of the long bones of 4–8-week-old C57BL/6 mice and were maintained in α-minimal essential medium (α-MEM) for 24 hours at 37°C, in 7% CO_2_, before separation with Ficoll gradient. To generate osteoclasts from BMMs, following Ficoll gradient separation, 1 × 10^5^ or 5 × 10^4^ cells, respectively, were plated in either 24-well or 48-well tissue culture plates. Cells were cultured in the presence of different concentrations and combinations of rM-CSF, RANKL, and IL-11 as indicated in individual experiments. The osteoclastogenesis cultures were stained for tartrate resistant acid phosphatase (TRAP) activity with a Leukocyte Acid Phosphatase kit (387-A) from Sigma. All assays were performed in triplicate and repeated at least three times. A representative view from each condition is shown.

### *In vitro* bone resorption assays

5 × 10^4^ BMMs were plated on bovine cortical bone slices in 24-well plates, and the cultures were treated with rM-CSF (10 ng/ml) and RANKL (100 ng/ml), or with IL-11, rM-CSF, or RANKL as detailed in each experiment. Cultures were maintained for 9 days to allow for bone resorption and then cells were removed from the bone slices with 0.25 M ammonium hydroxide and mechanical agitation. Bone slices were then subjected to scanning electron microscopy (SEM) using a Philips 515 SEM (Materials Engineering Department, University of Alabama at Birmingham). The percentage of the resorbed area was determined using ImageJ analysis software obtained from the National Institutes of Health.

### Statistical analysis

Osteoclastogenesis data are expressed as mean ± standard error (SE) of numbers of TRAP-positive cells. Cell viability assays are expressed as mean ± SE of numbers of viable cells on each day counted. Statistical significance was determined using Student’s *t* test, and *p* values less than 0.05 were considered significant.

## Results

### Breast cancer conditioned media are capable of supporting the development and/or survival of osteoclast progenitor cells

**Figure 1:**
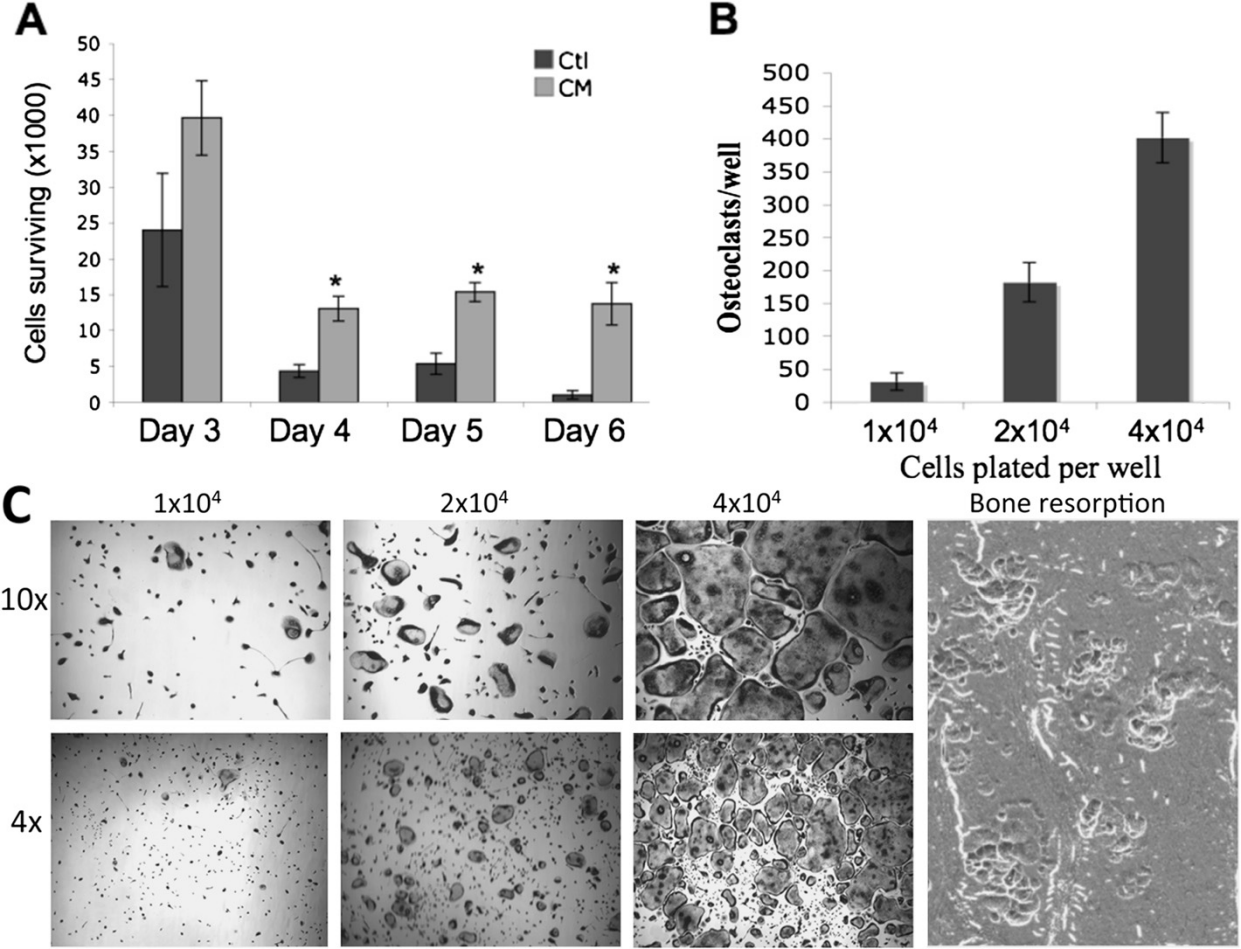
**Breast cancer conditioned media is capable of stimulating the development and/or survival of osteoclast progenitor cells.** (**A**) Bone marrow flushes were cultured in α-MEM (Ctl) or MDA-MB-231 conditioned α-MEM (CM) and surviving cells were counted at days 3, 4, 5, and 6. Data are expressed as a mean +/− S.E. *, p < 0.02. (**B**) On day 6, cells from the culture in breast cancer conditioned α-MEM were then seeded into 48 well plates at 1 × 10^4^, 2 × 10^4^, or 4 × 10^4^ cells per well and treated with 100 ng/ml RANKL and 10 ng/ml rM-CSF. Quantification of the osteoclastogenesis assays is shown in mean number of multinucleated TRAP-positive cells (>3 nuclei) per well. (**C**) Each condition had three replicates (wells) and was repeated 4 times. A representative area of the culture from each condition is shown (4× and 10× magnification). A separate set of cultures was continued 4 additional days to perform bone resorption assays. Resorption pits were then visualized by SEM. Magnification by SEM was 200x.

Given that previous studies showed that human breast cancer cell line MDA-MB-231 expresses IL-11 [13,20], we investigated whether MDA-MB-231 conditioned media are able to promote the development and/or survival of osteoclast progenitor cells in the whole bone marrow. Bone marrow flushes were cultured in regular media or breast cancer conditioned media, prepared from human breast cancer cell line MDA-MB-231, for varying number of days. As shown in Figure 1A, the cultures in the conditioned media had more cells than those in regular media. More importantly, we found that the cells from the cultures in the conditioned media were capable of forming functional osteoclasts in response to M-CSF and RANKL treatment, and that osteoclast number and morphology was dependent on density of progenitor cells plated (Figure 1B-C). These data indicate that breast cancer cells produce factors, presumably including IL-11, which are capable of stimulating the development and/or survival of osteoclast progenitor cells. Thus, these data suggest that breast cancer may enhance the extent of osteoclastogenesis by augmenting the pool of osteoclast progenitor cells.

### IL-11 promotes the development and/or survival of osteoclast progenitor cells

**Figure 2:**
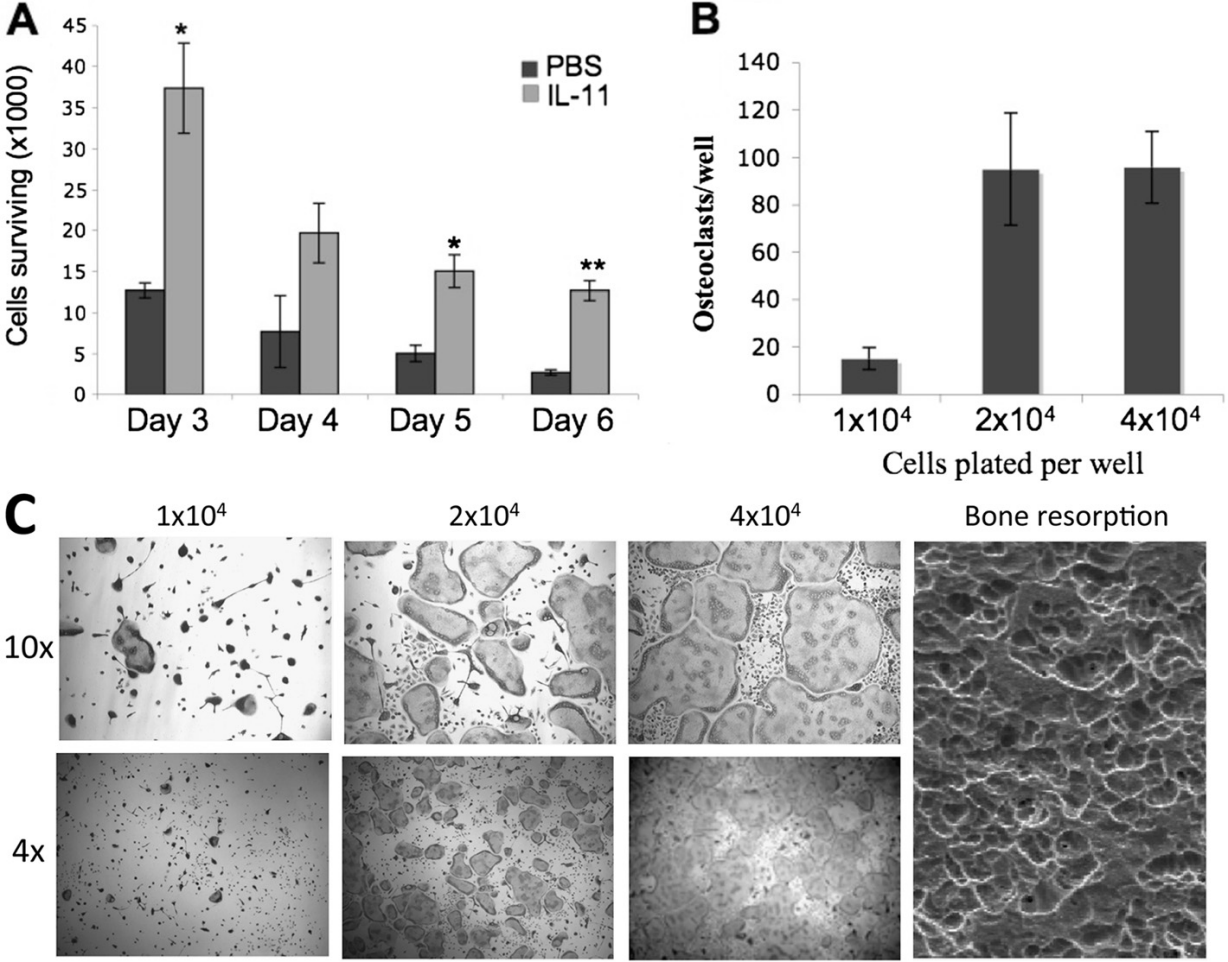
**IL-11 is able to promote the development and/or survival of osteoclast progenitor cells.** (**A**) Bone marrow flush cultured in α-MEM containing IL-11 (10 ng/ml) or equal volume of vehicle (PBS) and remaining surviving cells were counted at day 3, 4, 5, and 6. Data are expressed as a mean +/− S.E. *, p < 0.02, **, p < 0.002. (**B**) On day 6, IL-11-dependent bone marrow cells were then seeded into 48 well plates at 1 × 10^4^, 2 × 10^4^, or 4 × 10^4^ cells per well and treated with 100 ng/ml RANKL and 10 ng/ml rM-CSF. Quantification of the osteoclastogenesis assays is shown in mean number of multinucleated TRAP-positive cells (>3 nuclei) per well. (C) Each condition had three replicates (wells) and was repeated 4 times. A representative area of the culture from each condition is shown (4× and 10× magnification). A separate set of cultures was continued 4 additional days to perform bone resorption assays. Resorption pits were then visualized by SEM. Magnification by SEM was 200x.

Next, we examined whether recombinant IL-11 is able to replicate the results seen with breast cancer conditioned media (Figure 1). To do so, we cultured bone marrow flushes in α-MEM with or with IL-11 (10 ng/ml) for varying number of days. While most cells died in the cultures supplemented with vehicle (PBS), a significant number of cells remained alive and healthy in those treated with IL-11 (Figure 2A). Moreover, we found that the IL-11-dependent bone marrow cells were able to differentiate into functional osteoclasts in response to M-CSF and RANKL treatment (Figure 2B-C). Again, the osteoclast number and morphology was dependent on the density of the cells plated. These findings demonstrate that IL-11 is able to promote the development and/or survival of osteoclast progenitor cells.

### IL-11 neutralizing antibody reduces breast cancer conditioned media’s ability to promote the development and/or survival of osteoclast progenitor cells

**Figure 3:**
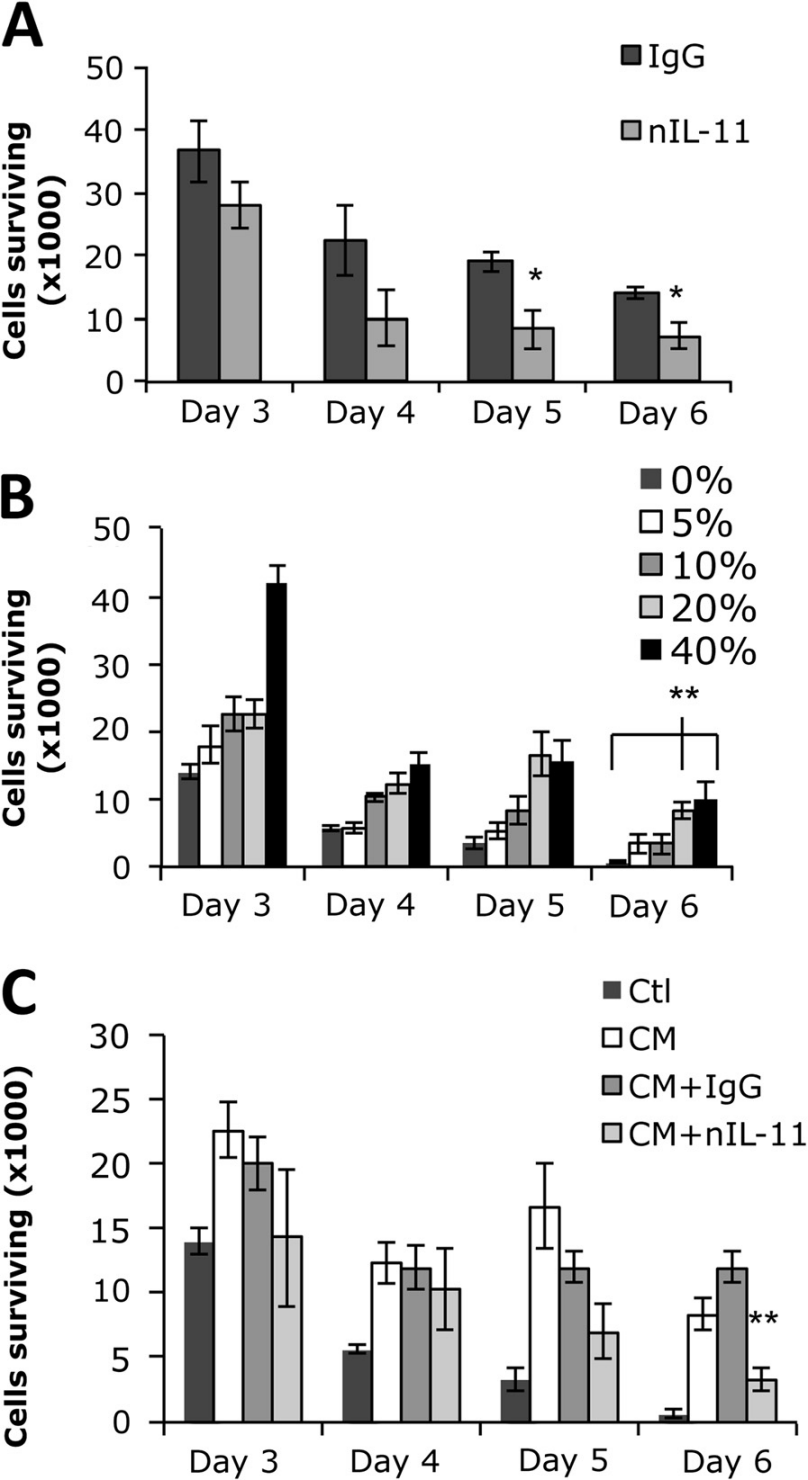
**IL-11 neutralizing antibody reduces breast cancer conditioned media’s ability to give rise to osteoclast progenitors.** (**A**) Bone marrow flushes were cultured in α-MEM containing IL-11 (10 ng/ml) with control IgG (IgG) or IL-11 neutralizing antibody (nIL-11, 2ug/ml) for 6 days. Surviving cells were counted at day 3, 4, 5, and 6. (**B**) Bone marrow flushes were cultured in α-MEM (0%) or α-MEM with increasing concentrations (5%, 10%, 20% or 40%) of MDA-MB-231 conditioned media for 6 days. Surviving cells were counted at day 3, 4, 5, and 6. (**C**) Bone marrow flushes were cultured in α-MEM (ctl), 20% MDA-MB-231 conditioned α-MEM (CM), 20% MDA-MB-231 conditioned α-MEM with control IgG (CM + IgG, 5 ug/ml), or 20% MDA-MB-231 conditioned α-MEM with IL-11 neutralizing antibody (CM + nIL-11, 5ug/ml). Surviving cells were counted at day 3, 4, 5, and 6. All data were repeated independently three times and are expressed as a mean +/− S.E, *, p < 0.05; **, p < 0.004.

To determine whether IL-11 is the predominant factor derived from MDA-MB-231 cells that stimulate the development and/or survival of osteoclast progenitors, we repeated the experiment shown in Figure 1 with an anti-human IL-11 neutralizing antibody. We first validated the neutralizing capability of the commercial IL-11 neutralizing antibody by culturing bone marrow flushes in α-MEM containing IL-11 (10 ng/ml) with control IgG or IL-11 neutralizing antibody (Figure 3A). Next, to determine the lowest optimal concentration of breast cancer conditioned media that could facilitate the development and/or survival of osteoclast progenitors, bone marrow flush cells were cultured in α-MEM or α-MEM with increasing concentrations of MDA-MB-231 conditioned media. There was no significant difference between using 20% or 40% breast cancer conditioned media, but both supported significantly more cells than 0, 5, or 10% (Figure 3B). Finally, bone marrow flushes cultured in 20% MDA-MB-231 breast cancer conditioned media were subjected to the IL-11 neutralizing antibody (5ug/ml) (Figure 3C). Our data demonstrated that the neutralizing IL-11 antibody significantly reduced the ability of the conditioned media to promote the development and/or survival of osteoclast progenitor cells, indicating that IL-11 is the predominant factor derived from MDA-MB-231 cells that stimulate the development and/or survival of osteoclast progenitors. This finding further suggests that certain breast cancers may increase the extent of osteoclastogenesis by expanding the pool of osteoclast progenitor cells via tumor-derived IL-11.

### IL-11 does not affect osteoclast survival

**Figure 4:**
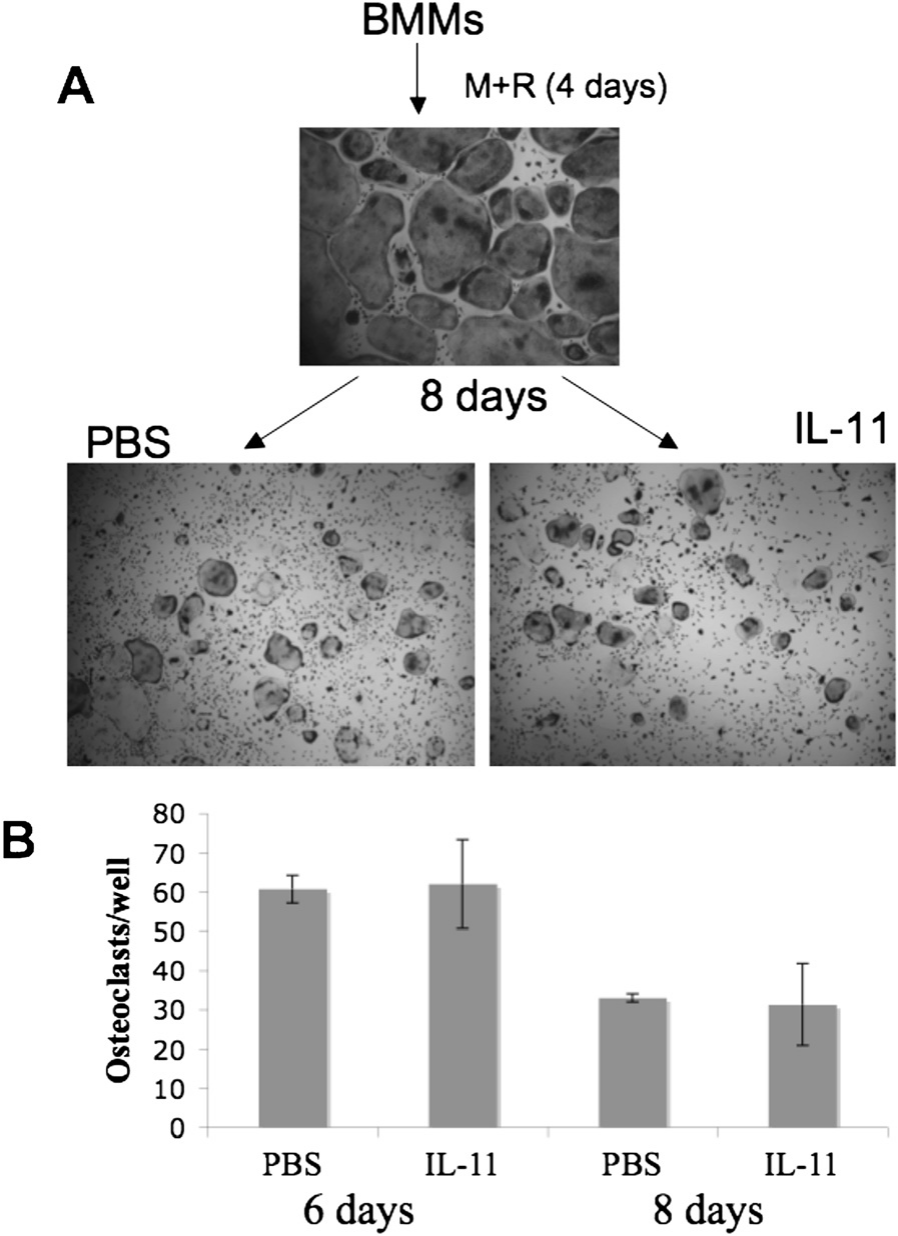
**IL-11 does not affect osteoclast survival.** (**A**) BMMs were cultured with rM-CSF (10 ng/ml) plus RANKL (100 ng/ml) for 4 days until sufficient osteoclasts were formed and then cultured with rM-CSF (10 ng/ml) and RANKL (100 ng/ml) with vehicle (PBS) or IL-11 (10 ng/ml) for 8 days in tissue culture dish. The cultures were then stained for TRAP activity. Each condition had three replicates (wells) and was repeated 4 times. A representative area of the culture from each condition is shown. (**B**) Quantification of the osteoclastogenesis assays is shown in mean number of multinucleated TRAP-positive cells (>3 nuclei) per well. Bars show averages ± S.D.

Given that our data have shows that IL-11 sustains a population of cells containing osteoclast precursors, we extended our study to address whether IL-11 exert any effect on the survival of mature osteoclasts. Towards this end, we treated BMMs with rM-CSF and RANKL for 4 days to promote osteoclast formation. Once osteoclasts formed, we removed the media containing rM-CSF and RANKL and added IL-11 or PBS (vehicle) to the cultures, which were continued for 8 additional days to determine IL-11’s effect on survival. The representative TRAP staining images of the osteoclast cultures treated with IL-11 for 8 days are shown in Figure 4A, while survived osteoclasts in the cultures treated with IL-11 were quantified at day 6 and day 8 (Figure 4B). At both day 6 and day 8, there was no statistically significant difference in osteoclast survival between the IL-11-treated cultures and the PBS-treated control cultures. Morphologically, the cultures also looked very similar. The data indicate that IL-11 does not play a role in osteoclast survival.

### IL-11 is unable to stimulate osteoclastogenesis in the absence of RANKL

**Figure 5:**
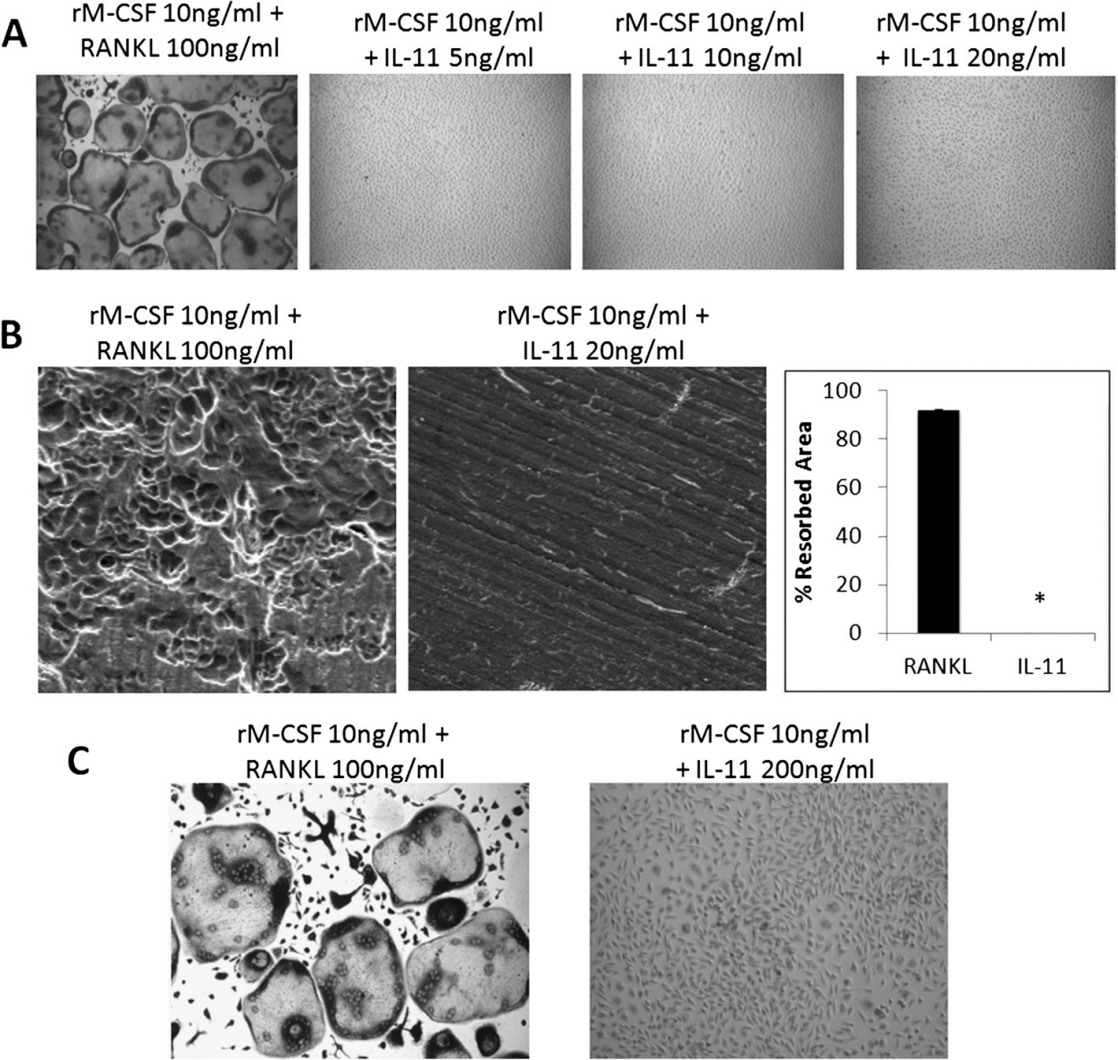
**IL-11 fails to stimulate osteoclastogenesis in the absence of RANKL.** (**A**) BMMs were cultured with rM-CSF (10 ng/ml) plus RANKL (100 ng/ml) as control or rM-CSF (10 ng/ml) plus IL-11 (5, 10 or 20 ng/ml) for 6 days in tissue culture dish. The cultures were then stained for TRAP activity. Each condition had three replicates (wells) and was repeated 4 times. A representative area of the culture from each condition is shown. (**B**) BMMS on bone slices were treated with rM-CSF (10 ;ng/ml) plus RANKL (100 ng/ml) as control or rM-CSF (10 ng/ml) plus IL-11 (20 ng/ml) for 9 days. Resorption pits were visualized by SEM. Magnification by SEM was 200x. Each resorption assay had two replicates (bone slices). Quantification of the bone resorption assays is shown, bars shown averaged ± S.E. *, p < 0.0001 (**C**) BMMs were cultured with rM-CSF (10 ng/ml) plus RANKL (100 ng/ml) as control or M-CSF (10 ng/ml) plus IL-11 (200 ng/ml) for 6 days in a tissue culture dish. The cultures were then stained for TRAP activity. Each condition had three replicates (wells) and was repeated 3 times. A representative area of the culture from each condition is shown.

The role of IL-11 in osteoclastogenesis remains unclear; while one study demonstrated that IL-11 is able to stimulate osteoclastogenesis independent of RANKL [15], another group showed that IL-11 cannot induce osteoclastogenesis unless marrow cells are co-cultured with calvaria cells [16], which may serve as a source of RANKL. To further investigate the role of IL-11 in osteoclastogenesis, we examined IL-11’s ability to induce osteoclastogenesis in the absence of RANKL. To this end, IL-11 was added at different concentrations (5, 10, and 20 ng/ml) along with 10 ng/ml rM-CSF to bone marrow macrophages, and then stained for TRAP activity on day 6 (Figure 5A). While confluent TRAP positive multinucleated osteoclasts were formed under control conditions of RANKL (100 ng/ml) and rM-CSF (10 ng/ml), none of the concentrations of IL-11 were sufficient to induce osteoclastogenesis in the absence of RANKL in tissue culture dishes. Furthermore, we performed bone resorption assays to determine whether IL-11 can promote osteoclast formation on bone slices. Our data reveal that IL-11 is incapable of stimulating functional osteoclasts on bone slices, as shown by the lack of resorption pits on the IL-11 treated bone slices (Figure 5B). To further address whether higher doses of IL-11 can promote osteoclastogenesis in the absence of RANKL, we repeated the experiment with 200 ng/ml IL-11. The data indicate that, even at concentrations as high as 200 ng/ml, IL-11 is still unable to stimulate osteoclastogenesis (Figure 5C). These findings indicate that IL-11 cannot promote osteoclast differentiation independent of RANKL.

### IL-11 cannot stimulate osteoclastogenesis even with low levels of RANKL

**Figure 6:**
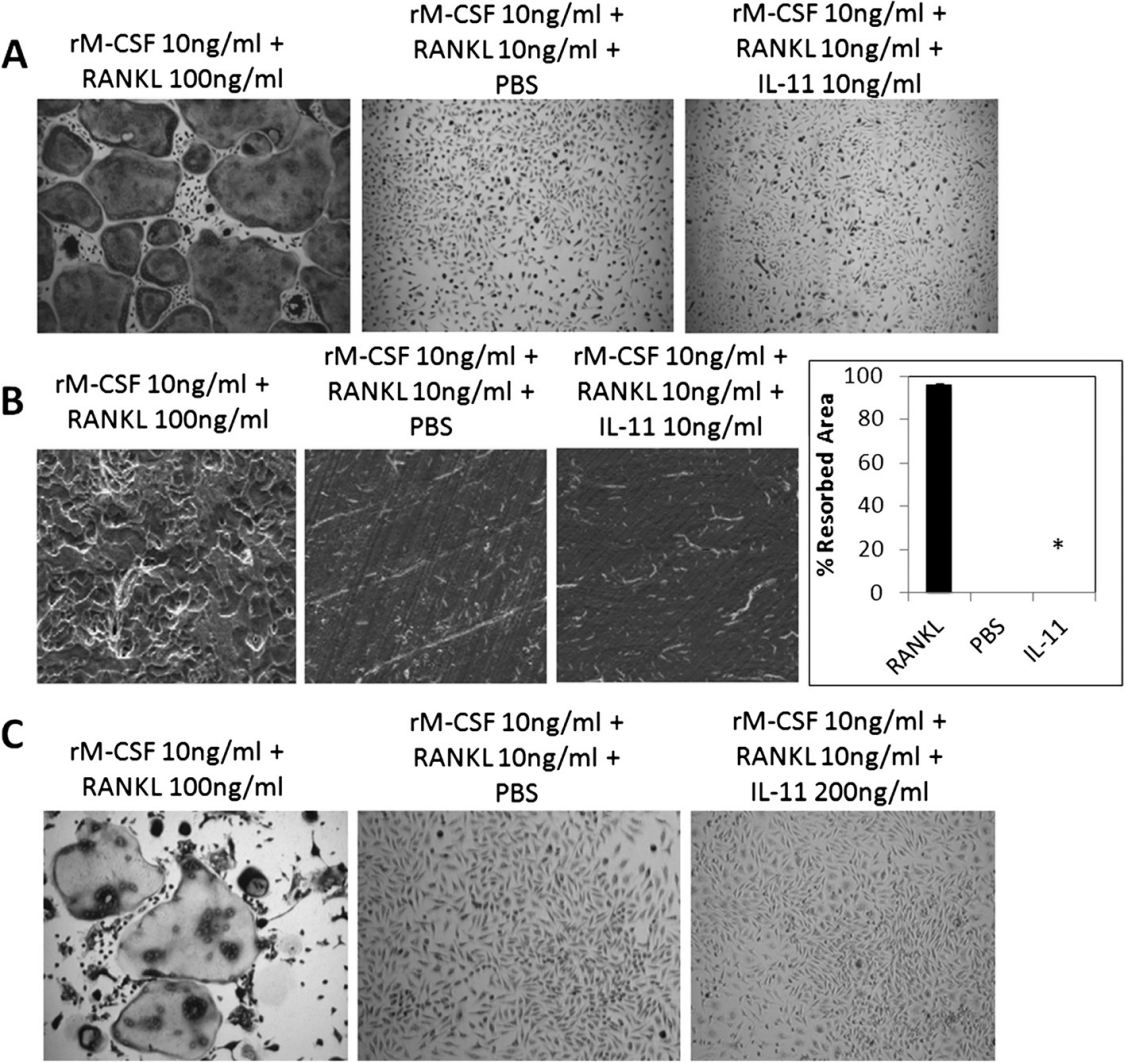
**IL-11 fails to stimulate osteoclastogenesis even with permissive level of RANKL.** (**A**) BMMs were cultured with rM-CSF (10 ng/ml) plus RANKL (10 ng/ml) with or without IL-11 (10 ng/ml) for 6 days in tissue culture dish. The cultures were then stained for TRAP activity. Each condition had three replicates (wells) and was repeated 4 times. A representative area of the culture from each condition is shown. (**B**) BMMs on bone slices were treated with the same conditions, but cultured for 9 days and resorption pits were then visualized by SEM. Magnification by SEM was 200x. Each resorption assay had two replicates (bone slices) Quantification of the bone resorption assays is shown, bars shown averaged ± S.E. *, p < 0.0001 (**C**) BMMs were cultured with rM-CSF (10 ng/ml) plus RANKL (100 ng/ml) as control or rM-CSF (10 ng/ml) with sub-optimal levels of RANKL (10 ng/ml) with or without IL-11 (200 ng/ml) for 6 days in a tissue culture dish. The cultures were then stained for TRAP activity. Each condition had three replicates (wells) and was repeated 3 times. A representative area of the culture from each condition is shown.

We and others have demonstrated that although several cytokines such as IL-1 and tumor necrosis factor α (TNF-α) cannot promote osteoclastogenesis in the absence of RANKL, they are able to do so in the presence of permissive levels of RANKL [21-26]. So we next investigated if IL-11 can stimulate osteoclastogenesis in the presence of low levels of RANKL. BMMs were cultured with rM-CSF (10 ng/ml) plus RANKL (10 ng/ml) with or without IL-11 (10 ng/ml) for 6 days in tissue culture dishes and then stained for TRAP activity (Figure 6A). We found that IL-11 was not able to induce osteoclastogenesis with low levels of RANKL in tissue culture dishes. The assay was repeated on bone slices and the bone resorption assays showed that IL-11 failed to promote the formation of functional osteoclasts on bone slices in the presence of 10 ng/ml RANKL, as shown by the lack of resorption pits. (Figure 6B). To further address whether higher doses of IL-11 can promote osteoclastogenesis in the low levels of RANKL, we repeated the experiment with 200 ng/ml IL-11. The data indicate that, even at concentrations as high as 200 ng/ml, IL-11 is still unable to stimulate osteoclastogenesis in the presence of low levels of RANKL (Figure 6C). Taken together, we conclude that IL-11, unlike IL-1 and TNF-α, is incapable of stimulating osteoclastogenesis even in the presence of low levels of RANKL.

### IL-11 is incapable of stimulating osteoclastogenesis from RANKL-primed BMMs

**Figure 7:**
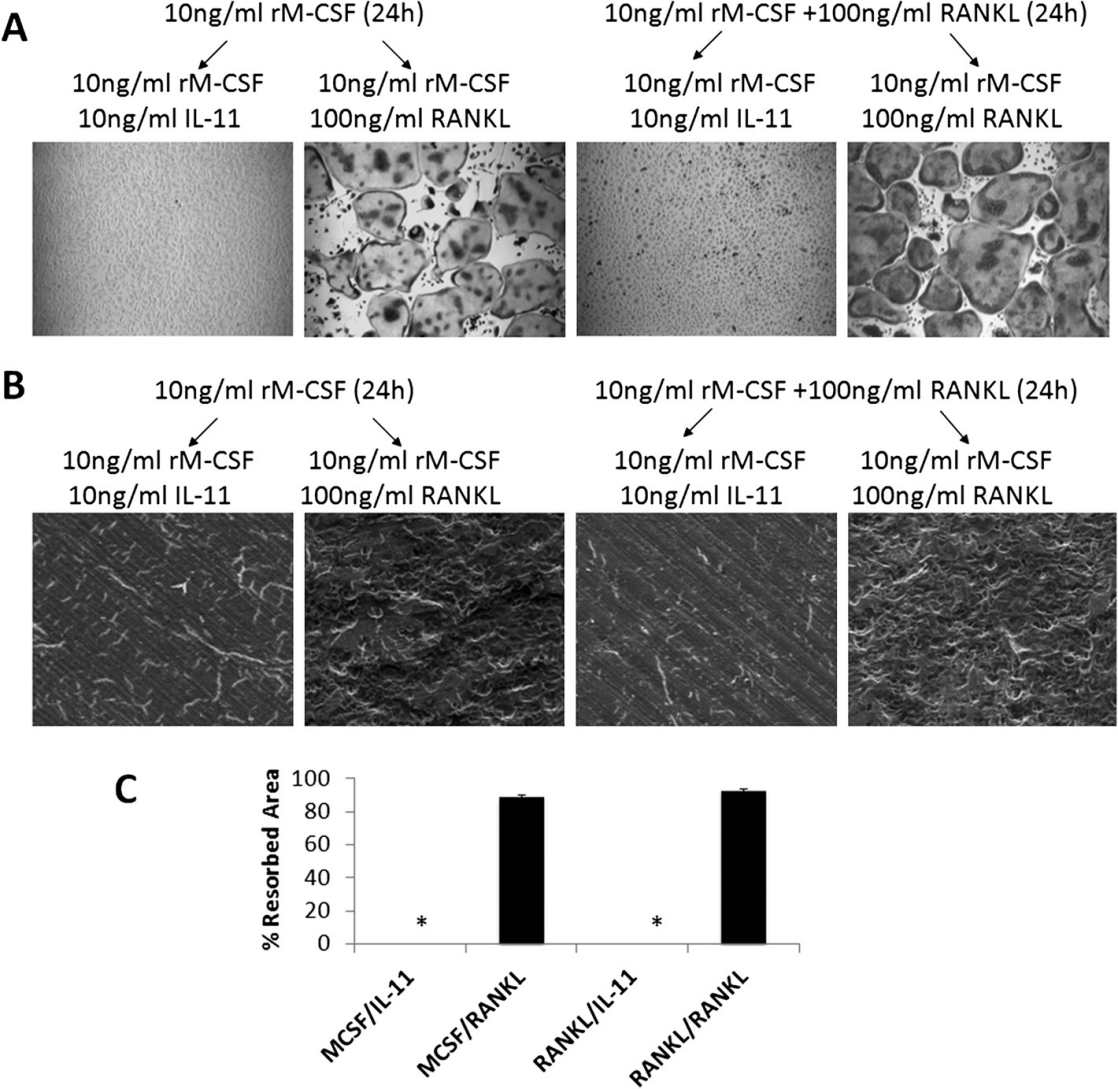
**IL-11 fails to stimulate osteoclastogenesis even when BMMs are primed with RANKL for 24 hours.** (**A**) BMMs were pretreated with rM-CSF (10 ng/ml) or rM-CSF (10 ng/ml) and RANKL (100 ng/ml) for 24 hours and then cultured with rM-CSF (10 ng/ml) plus IL-11 (10 ng/ml) or RANKL (100 ng/ml) for 4 days in tissue culture dish. The cultures were then stained for TRAP activity. Each condition had three replicates (wells) and was repeated 4 times. A representative area of the culture from each condition is shown. (**B**) BMMS on bone slices were treated with the same conditions, but cultured for 9 days. Resorption pits were then visualized by SEM. Magnification by SEM was 200x. Each resorption assay had two replicates (bone slices). (**C**) Quantification of the bone resorption assays is shown, bars shown averaged ± S.E. *, p < 0.0001.

We and others have also shown that IL-1 and TNF-α can also promote osteoclastogenesis from RANKL-primed BMMs [21-26]. To determine whether IL-11 can function in osteoclastogenesis in this manner, BMMs were pretreated for 24 hours with or without RANKL in the presence of rM-CSF in tissue culture dishes or on bone slices. After 24 hours, the media was removed and replaced with media containing rM-CSF with either IL-11 or RANKL, and the cultures were continued for 4 days (Figure 7). The assays demonstrated that IL-11 was unable to stimulate osteoclastogenesis from RANKL-primed BMMs in tissue culture dishes (Figure 7A) or on bone slice (Figure 7, B and C).

## Discussion

Since the initial study showing the expression of IL-11 in breast tumor tissues more than 25 years ago [27], numerous investigations have been subsequently undertaken to address the regulation and pathological significance of IL-11 expression in breast cancer and, in particular, in the tumor-induced osteolysis [5,13,28-34]. Collectively, these studies have led to two important observations: a) IL-11 is not only expressed in a significant number of breast cancers but also has the potential to serve as a prognostic factor in human breast cancer, and b) IL-11 plays an important role in breast cancer-mediated osteolysis by promoting osteoclastogenesis and bone resorption. Notably, several studies have demonstrated that breast tumor cells can also target osteoblasts to stimulate their production of IL-11 [11,17], further increasing IL-11 concentrations in the bone microenvironment. Therefore, elucidation of the molecular mechanism by which IL-11 increases osteoclastogenesis and bone resorption in breast cancer bone metastasis may help guide development of effective drugs and/or therapeutic regimens for preventing and treating breast cancer-induced osteolysis.

Early studies on the role of IL-11 in osteoclast formation and function involved the use of the co-culture system containing bone marrow cells and calvarial osteoblasts [16,35]; the key finding of these early investigations was that IL-11-mediated osteoclastogenesis requires the presence of osteoblasts, but the precise reason for the dependence of IL-11-mediated osteoclastogenesis on osteoblasts was not fully understood. After the discovery of the RANKL/RANK/OPG system in the late 1990s, it then became clear that osteoblasts in the co-culture system primarily serve as a source of RANKL and IL-11 stimulates osteoblasts to produce RANKL [36,37]. This led to the notion that IL-11 can promote osteoclastogenesis indirectly by stimulation osteoblast production of RANKL. On the other hand, it was shown that osteoclasts express IL-11R [35], suggesting that IL-11 may also directly target osteoclasts and/or its precursors to regulate osteoclast formation and/or function. Intriguingly, one study demonstrated that IL-11 directly target osteoclast precursors to stimulate osteoclastogenesis and it does so independent of RANKL [15]. However, this finding is inconsistent with the early studies showing that IL-11-mediated osteoclastogenesis requires the presence of osteoblasts, which is a known source of RANKL.

In this work, we independently carried out a series of *in vitro* studies to further address the role of IL-11 in osteoclastogenesis. First we determined that the conditioned media of MDA-MB-231, a breast cancer cell line expressing IL-11 [13,20], gave rise to a population of cells which can form osteoclasts in response to RANKL and M-CSF treatment (Figure 1), indicating that IL-11 may play an important role in osteoclastogenesis by regulating the development and/or survival of osteoclast progenitor cells. Because the MDA-MB-231 also secrete other factors that play a role in osteoclastogenesis it was necessary to look specifically at IL-11 function. Importantly, the ability of the breast cancer conditioned media to generate a population of osteoclast progenitor cells was significantly inhibited by a neutralizing anti-IL-11 antibody (Figure 3). These findings suggest that tumor-derived IL-11 may increase the extent of osteoclastogenesis by promoting the development of a population of osteoclast progenitor cells. To verify the specificity of IL-11, we found that culturing of murine bone marrow cells with IL-11 for 6 days is able to give rise to a pool of osteoclast progenitor cells (Figure 2).

We then investigated other ways that IL-11 may play a role in osteoclastogenesis. We found that IL-11 does not exert any effect on osteoclast survival (Figure 4). We then examined if IL-11 is able to promote osteoclast formation in the absence of RANKL and our data demonstrate that IL-11 cannot induce osteoclastogenesis in tissue culture dishes or on bone slices in the absence of RANKL (Figure 5). We and others have demonstrated that while IL-1 and TNF-α cannot promote osteoclastogenesis in the absence of RANKL, they can do so with suboptimal levels of RANKL or from RANKL-pretreated BMMs [21-26]. As such, we then investigated whether IL-11 can act in a similar manner. Our data show that IL-11 is not able to promote osteoclastogenesis in the presence of suboptimal levels of RANKL (Figure 6) or from RANKL-pretreated BMMs (Figure 7).

Based on these new findings and those reported previously [16,34,36,37], we propose that IL-11-expressing breast cancer cells cause increased osteoclast formation and bone resorption by two distinct mechanisms: a) the tumor cells produce IL-11 which in turn stimulate the production of RANKL by stromal cells/osteoblasts in the bone microenvironment, and b) tumor cell-derived IL-11 also augments the pool of osteoclast progenitor cells to increase the extent of osteoclastogenesis. Therefore, our work has led to a better understanding of the action of IL-11 in breast cancer-induced osteolysis. However, the precise mechanism by which IL-11 promotes the development of a population of osteoclast progenitor cells remains unclear. While it is possible that IL-11 does so by stimulating the differentiation, proliferation and/or survival of osteoclast progenitor cells, this cytokine may exert the impact on osteoclast progenitor cell population indirectly through other cell types in the bone marrow. Further studies are needed to elucidate how exactly IL-11 promote the development of a pool of osteoclast progenitor cells.

Moreover, our new data may help guide the development of better therapeutic regimens for preventing and treating breast cancer-induced osteolysis. Particularly, denosumab, a humanized anti-RANKL developed by Amgen Inc, has been approved by the FDA to treat breast cancer-mediated osteolysis. For IL-11 positive tumors, denosumab may be effective only in blocking the RANKL-dependent action of IL-11. In contrast, it is likely that an efficient inhibition of IL-11 can block the IL-11-mediated increase of the pool of osteoclast progenitor cells as well as the RANKL-dependent pathway, thus having the potential to give rise to better efficacy. Future animal model studies need to be undertaken to address the therapeutic potential of targeting IL-11.

## Conclusions

In conclusion, these studies demonstrate that IL-11 exerts its effect on osteoclastogenesis primarily by targeting osteoclast progenitor cells, specifically through promoting the development and/or survival of osteoclast progenitor cells. Moreover, we show that MDA-MB-231 breast cancer cells are able to stimulate the development and/or survival of osteoclast progenitor cells and IL-11 is the predominant factor derived from MDA-MB-231 cells that is responsible. This suggests that some breast cancers may increase the extent of osteoclastogenesis by augmenting the pool of osteoclast progenitor cells via tumor-derived IL-11. Importantly, these findings have not only provided a better understanding of the role of IL-11 in breast cancer bone metastasis but also laid a foundation for future investigations to address therapeutic targeting of IL-11 for treating and preventing breast cancer induced osteolysis.

## Abbreviations

α-MEM: α-Minimal essential medium; BMMs: Bone marrow macrophages; IL-11: Interleukin 11; M-CSF: Monocyte/macrophage-colony stimulating factor; RANKL: Receptor activator of nuclear factor κB ligand; rM-CSF: Recombinant M-CSF; SEM: Scanning electron microscopy; TRAP: Tartrate resistant acid phosphatase; TNF-α: Tumor necrosis factor α; IL-1: Interleukin 1; IL-6: Interleukin 6.

## Competing interests

The authors declare that they have no competing interests.

## Authors’ contributions

All authors read and approved the final manuscript. EM developed the idea, performed the experiments, analyzed the data, and prepared the manuscript. HH and HCP provided technical assistance. XF initially conceived the idea, and participated in the experimental design and manuscript preparation.

## Pre-publication history

The pre-publication history for this paper can be accessed here:

http://www.biomedcentral.com/1471-2407/13/16/prepub
